# Genomic characterization of *Comamonas kerstersii* isolated from diarrheal patients in Bangladesh

**DOI:** 10.1371/journal.pone.0346980

**Published:** 2026-04-10

**Authors:** Noshin Ibnat Rib, Fariza Shams, Fahad Khan, Senzuti Sharmin, Sakib Abrar Hossain, Abdus Sadique, Jahidul Alam, Pronoy Debnath, Arman Hossain, Aura Rahman, Syeda Naushin Tabassum, Tahrima Saiha Huq, Muhammad Maqsud Hossain

**Affiliations:** 1 NSU Genome Research Institute (NGRI), North South University, Dhaka, Bangladesh; 2 Department of Biochemistry and Microbiology, North South University, Bangladesh; Texas A&M University, UNITED STATES OF AMERICA

## Abstract

This study marks the first identification and genomic characterization of *Comamonas kerstersii* isolates from diarrheal patients in Bangladesh. We carried out the whole genome sequencing of three *C. kerstersii isolates* to analyze genomic features using bioinformatics tools. We hypothesize that *C. kerstersii* can contribute to the diarrheal disease process through indirect mechanisms, potentially by interacting synergistically with other enteric pathogens such as *Vibrio cholerae* (both O1 and non-O1 serogroups). The presence of diverse virulence factors, including type IV pili, type VI secretion systems, chemotaxis proteins, and toxin genes such as *zot* and RTX, suggests a capacity for adhesion, motility, and immune evasion. Notably, genomic analyses indicate that *C. kerstersii* shares several offensive and defensive virulence factors with other pathogenic *Comamonas* spp, including mechanisms for biofilm formation, nutrient acquisition, and stress tolerance. These factors, combined with antimicrobial resistance genes identified genes – *aph(6)-Id, aph(3”)-Ib, mph(E), mph(F), msr(E), sul2,* and *tet(A),* may enhance survival and adaptability of *C. kerstersii* in the gut environment, potentially augmenting the pathogenicity of co-infecting diarrheal pathogens. These initial findings highlight the need for extensive genomic surveillance across diarrheal patients, along with further investigation into molecular interactions with co-pathogens that could reveal novel pathways influencing diarrheal disease outcomes.

## Introduction

The increasing number of persistent infections caused by relatively unknown non-fermenting gram-negative rods is a crucial concern in healthcare environments [1]. These emerging pathogens, which include species from the *Comamonas* genus, are opportunistic and pose severe risk to global health [2]. One little-known species in the *Comamonas* genus is *Comamonas kerstersii* [3]. *C. kerstersii* was first recognized as a distinct species in 2003, alongside *Comamonas aquatica* and *Comamonas terrigena* [3–6].

This species belongs to rRNA homology group III and is characterized by the presence of a unique 23S rRNA gene and a distinct 16S rRNA gene sequence [7,8]. *C. kerstersii* was initially included in the *C. terrigena* complex [9]. Subsequent taxonomic studies by Willems et al. and Wauters, et al and demonstrated that *C. terrigena* contains 3 different DNA groups [4,10]. Based on the 16S rRNA sequencing and DNA-DNA hybridization analyses, DNA group I retained the classification *C. terrigena,* whereas DNA groups II and III were reclassified as *C. aquatica* and *C. kerstersii,* respectively [4].

*C. kerstersi* species are non-fermenting, gram-negative, rod-shaped bacillus [9]. The smooth-surfaced and non-pigmented colonies have a diameter of 1.5 mm on blood agar when grown under an ambient aerobic environment for 48 hours of incubation [9,11]. *C. kerstersii* has previously been considered nonpathogenic. It is extensively found in soil, plants, and water with the ability to withstand aquatic environments [3]. Although once considered nonpathogenic, *C. kerstersii* is now increasingly acknowledged as an environmental organism with opportunistic pathogenic potential, especially in polymicrobial intra-abdominal infections. In recent years, the species have been found in several clinical samples like abdominal fluid, peritoneal fluid, blood, urine, stool and recently in sputum [5,6,9,11–16]. Reported infections include conditions like intra-abdominal including appendicitis, peritonitis, diverticulosis, and a few extra abdominal cases, including urinary tract infection, bacteremia and pneumonia, have been reported [5,6,9,11–16]. Most infections associated with *C. kerstersii have* been polymicrobial in nature [2,17].

Species of the genus *Comamonas* are associated with certain human infections and exhibit diversified virulence factors [15,18]. Studies have shown that *C. aquatica*, *C. terrigena*, and *C. kerstersii* form a distinct cluster, sharing common virulence genes involved in bacterial motility, adhesion, and chemotaxis signaling [19]. These include adhesin biosynthesis genes like *IlpA* and *Hsp60*, biofilm-forming components, hemolysin genes, and stress response proteins such as *ClpEP* and *SodB*, which contribute to cellular defense [19].

In Bangladesh, diarrheal diseases are driven by a spectrum of enteric pathogens including *V. cholerae, Shigella* spp*., Salmonella* spp., rotavirus, entero-toxigenic *Escherichia coli* (ETEC), with ETEC and *Vibrio cholerae* O1 together accounting for nearly 50% of annual cases and exhibiting varying incidence and severity across age groups [20,21]. The studies report that mixed infections contribute to 12% to 26% of acute watery diarrhea cases [20,21]. Additionally, a significant proportion of diarrheal cases ranging from 36.5% to 51% across different surveillance periods have no identifiable enteric pathogen using conventional laboratory methods [20,22]. While this does not establish the presence of emerging or novel bacterial agents, it highlights the limitations of standard detection methods. Biswas et al. reported that over a period of two years, pure growth of *C. kerstersii* was isolated from 27 fecal samples submitted for diarrhea investigation and suggested that environmental exposure and subsequent carriage within the bowel may be more common than currently assumed [23]. Furthermore, *Comamona*s spp., including *C. aquatica* and *C. testosteroni*, have been detected in stool samples from patients with gastrointestinal symptoms like diarrhea and abdominal pain [24].

While direct causality remains unprovenit is still unclear if *Comamonas* spp. directly cause these symptoms, the repeated detection of *C. kerstersii* and related *comamonas* spp. in stool samples suggests a possible opportunistic role in diarrheal diseases specially in polymicrobial contexts. We hypothesize that *C. kerstersii* could participate in the pathogenesis of diarrheal diseases indirectly, possibly via synergistic relationships with other enteric pathogens. The presence of various offensive and defensive virulence factors including mechanisms for biofilm formation, nutrient acquisition, and stress tolerance in *C. kerstersii* and other *Comamonas* spp. [19], further indicates its capacity to survive and adapt in the gut environment and potentially augmenting the pathogenicity of co-infecting diarrheal pathogens.

Given its widespread distribution and increasing detection in clinical samples, it is important to investigate the genomic characteristics and pathogenic potential of *C. kerstersii*, particularly in regions like Bangladesh where data are limited. This study presents the first comprehensive genomic analysis of *C. kerstersii* isolates from diarrheal patients in Bangladesh. It provides insights into their antimicrobial resistance profiles and potential virulence factors, contributing to a better understanding of the role of this emerging organism in gastrointestinal infections.

## Materials and methods

### Sample collection, bacterial isolation and molecular identification

From 11 March 2020–12 November 2020, 17 stool samples were collected from patients presenting with acute gastroenteritis at Dhaka Central International Medical College & Hospital (DCIMCH) under institutional ethical approval (2020/OR-NSU/IRB/1205), with the initial aim of isolating *V. cholerae*. Prior to sample collection, each patient was informed of the study's purpose and the voluntary nature of participation. All individuals provided verbal informed consent prior to sample collection. The consent procedure was approved by North South University's Institutional Review Board (IRB). Verbal consent was documented by the attending clinician or research staff on the sample collection record, and the process was witnessed by a member of the clinical team. To protect the confidentiality of participants, no personally identifiable information was recorded.

Samples were transported in 5 mL Cary-Blair transport medium at 4°C. For enrichment, 3 mL of each sample was incubated in alkaline peptone water (APW) at 37°C for 6–8 hours. Following enrichment, aliquots were streaked onto thiosulfate-citrate-bile salts-sucrose (TCBS) agar and tellurite taurocholate gelatin agar (TTGA) plates, which were incubated overnight at 37°C. Yellow colonies on TCBS and black-centered colonies on TTGA—presumptively identified as *V. cholerae* based on colony morphology—were sub cultured onto gelatin agar (GA) for further screening.

Subsequent Gram staining and standard biochemical tests were performed for characterization of isolates. Of the 17 presumptive *Vibrio* isolates, 14 demonstrated gelatin hydrolysis on GA, consistent with *V. cholerae* characteristics. However, three isolates (NG13, NG14, and NG17) did not show gelatin hydrolysis and exhibited biochemical profiles inconsistent with *V. cholerae*. These three isolates were therefore subjected to 16S rRNA gene sequencing for definitive identification. Sequencing results confirmed that they belonged to the *Comamonas* genus, indicating initial misidentification based on conventional methods. A summary of their distinguishing biochemical profiles is provided in Table 2.

### Genome sequencing, assembly, and annotation

Isolates were inoculated in lysogeny broth (LB) and grown at 37°C overnight for DNA extraction. Genomic DNA was extracted using QIAamp® DNA Mini Kit (QIAGEN, Hilden, Germany) according to the manufacturer's protocol. Library preparation and sequencing of 3 selected strains were carried out at Genome Research Institute of North South University (NGRI), Bangladesh. High molecular weight genomic DNA of the strains was used to prepare libraries using a Nextera DNA Library Prep Kit and employed for 250-bp paired-end whole genome sequencing with Illumina® MiSeq platform following the instruction provided by the manufacturer. High-quality paired-end Illumina sequencing data (Q  ≥  30) were obtained for the bioinformatic analysis. Quality control was performed using Quast [25] on the sequencing data to ensure the integrity and accuracy of the information. SPAdes genome assembly software (version 3.15.5) [26] was used for the de novo assembly of the genome with 20x to 30x coverage and particular filters employed to lower the number of mismatches and short indels, improving the quality of the generated contigs. The PROKKA pipeline (version 45) [27] was employed for annotation to decipher the genetic content and functional elements encoded within the assembled contigs. Rapid Annotation using Subsystem Technology (RAST) [28], an automated service for annotating genomes of bacteria and archaea, was also employed for annotation.

### Genomic feature determination

Nine genomes (S2 File) of *C. kerstersii* were obtained from the National Center for Biotechnology Information (NCBI). The obtained genomes were annotated using the PROKKA pipeline [27]. The core genome alignment was generated using Roary [29] and then used in FastTree [30] to construct a midpoint rooting phylogenetic tree and visualized using FigTree. Assembled genomes were visualized using the BLAST ring image generator (BRIG) [31]. Antimicrobial resistance genes were identified utilizing the Center for Genomic Epidemiology (CGE) based tool ResFinder (version 4.1) [32]. In this study, the Virulence Factor Database (VFDB) (http://www.mgc.ac.cn/VFs/) has been used to identify and analyze virulence factors associated with the studied strains [33]. Identification of plasmids was conducted employing PlasmidFinder [34] and phage regions using PHASTEST [35] and Phigaro [36] respectively.

### Host pathogen protein-protein interaction (PPI) network construction

Amino acid sequences from the three *C. kerstersii* were clustered using MMseqs2 (Steinegger & Söding, 2017) (version 13.45111) with 90% identity and 80% coverage thresholds. To construct a host–pathogen interaction (HPI) network, 9,333 human–bacterial PPIs were retrieved from the PHISTO and 9,031 unique interactions were retained after removing duplicates. Corresponding bacterial protein sequences were then obtained from UniProt [37]. Clustered *Comamonas* proteins were aligned to these sequences using BLASTp [38] (NCBI BLAST+ v2.5.0) and the resulting predicted *Comamonas*–human PPI network was visualized and analyzed in NetworkX (version 2.8.8), identifying the top 10 hub proteins based on degree centrality. These hub proteins were subsequently functionally annotated using EggNOG-mapper v2 [39] to predict their biological roles through orthology-based inference. To further investigate host response, KEGG pathway enrichment analysis was performed on 1,360 human interacting proteins using the R package clusterProfiler (version 4.12.6) [40], applying a p-value and q-value cutoff of 0.05 with Benjamini-Hochberg correction. The top 15 significantly enriched pathways (FDR < 0.05) were selected, revealing key host cellular processes potentially targeted during *Comamonas* infection. It is important to note that the host–pathogen interactions reported here are computational predictions based on sequence similarity to experimentally validated interactions in the PHISTO database and have not been experimentally validated in this study.

## Results

### Clinical characteristics and bacterial identification

Three patients were found to carry bacterial isolates that were later identified as *C. kerstersii* (NG13, NG14, and NG17). All three presented with acute gastrointestinal symptoms, including watery diarrhea (three or more episodes per day) and vomiting, along with varying degrees of dehydration. Their demographic details, clinical history, presenting symptoms, and treatment are summarized in Table 1.

**Table 1:** Demographics, clinical characteristics, and treatment of affected patients.

Patient ID	Age	Sex	Antibiotic Usage	Probiotic Consumption	Water Consumption	Symptoms	Treatment
1	19	Male	Yes	Curd	Boiled water	Watery diarrhea (≥3 times/day), vomiting, dehydration, oliguria	Oral and IV (intravenous) saline administered
2	40	Female	No	Curd	Municipal water	Watery diarrhea (≥3 times/day), vomiting, dehydration, anuria for ~12 hours	Oral and IV saline administered
3	24	Female	Yes	Curd	Boiled water	Watery diarrhea (≥3 times/day), vomiting, severe dehydration, stomachache, oliguria	Oral and IV saline administered

Patient 1 (sample NG13) was a 19-year-old male who consumed boiled water and reported probiotic intake in the form of curd. He experienced moderate dehydration accompanied by oliguria. He was treated with both oral and intravenous saline, as well as antibiotics. Patient 2 (sample NG14) was a 40-year-old female who consumed municipal (untreated) water and consumed curd. She presented with similar gastrointestinal symptoms but experienced more severe dehydration, including anuria lasting approximately 12 hours. She was managed with fluid and electrolyte replacement without antibiotic intervention. Patient 3 (sample NG17) was a 24-year-old female who consumed boiled water and curd. Her symptoms included diarrhea, vomiting, stomach pain, and oliguria, with signs of severe dehydration. Like Patient 1, she was treated with antibiotics in addition to oral and IV saline.

Initially, the bacterial isolates from these patients were presumptively identified as *V. cholerae* based on colony morphology on selective media. However, biochemical testing showed that all three isolates were oxidase- and catalase-positive, motile, and capable of reducing nitrate and hydrolyzing trypsin. Importantly, none of the isolates demonstrated gelatin hydrolysis, which is typically associated with *V. cholerae*. They also tested negative for acid production from glucose, urease activity, hydrogen sulfide (H₂S) production, and indole formation. Given the inconsistency with typical *V. cholerae* profiles, 16S rRNA gene sequencing was performed and confirmed the identity of all three isolates as *Comamonas spp*. The detailed biochemical characteristics of these isolates are provided in Table 2.

**Table 2:** Biochemical test results of the three *C. kerstersii* isolates.

Test Name	NG13	NG14	NG17
Oxidase	+	+	+
Catalase	+	+	+
Motility	+	+	+
Gelatin Hydrolysis	–	–	–
Nitrate Reduction	+	+	+
Trypsin Hydrolysis	+	+	+
Acid from Glucose	–	–	–
Urease	–	–	–
H_2_S Production	–	–	–
Indole	–	–	–

### Genomics features

**Fig 1:**
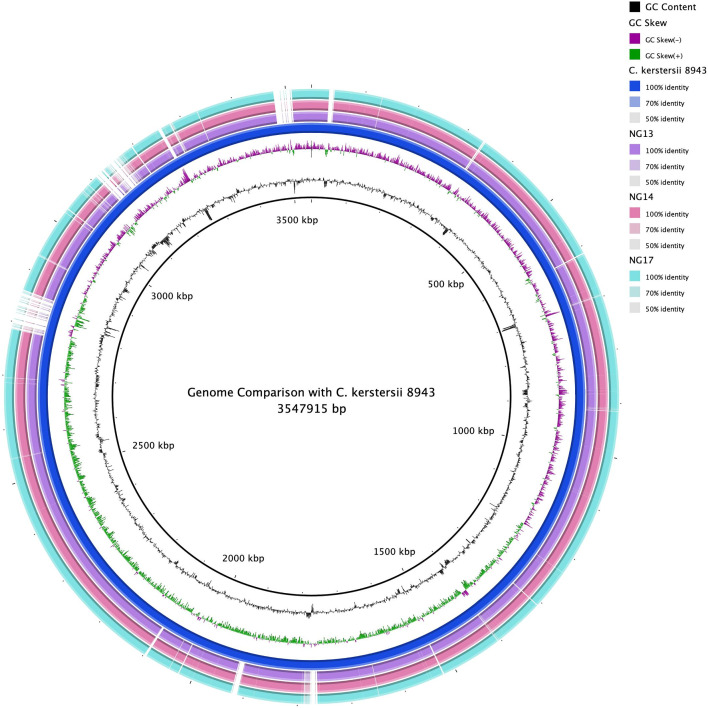
BRIG-obtained circular genomic maps of *C. kerstersii* strains. Circular representation of the genomes of the strains NG13, NG14, and NG17 showcasing coding sequences (CDS), GC content and GC skew. The homology rate is indicated by color saturation; blanks indicate no resemblance. The blue circle represents the reference genome *C. kerstersii* 8943.

Draft genome assemblies of *Comamonas* strains—NG13, NG14, and NG17 yielded 50, 68, and 81 contigs respectively. The assembled genome sizes were approximately 3.48 Mbp for each strain, with a consistent GC content of around 59.6% (Fig 1).

Gene prediction and annotation revealed a similar number of coding sequences (CDS) across all three isolates, ranging from 3,211–3,215. The number of tRNA genes varied slightly, with NG13 and NG17 containing 94 tRNAs and NG14 containing 97. All strains carried 7 rRNA genes and 1 transfer-messenger RNA (tmRNA). The N50 values, indicating assembly continuity, ranged from 181,861 bp (NG13) to 284,992 bp (NG14). A detailed summary of the genomic features is presented in Table 3.

**Table 3:** Genome assembly and annotation statistics of the three *C. kerstersii* isolates.

	NG13	NG14	NG17
Genome Size (appx)	34, 806, 663	34, 90, 070	34, 93, 044
No. of contigs	50	68	81
GC (%)	59.60	59.60	59.59
No. of CDS	3211	3215	3213
No. of tRNA	94	97	94
No. of rRNA	7	7	7
No. of tmRNA	1	1	1
N50 (bp)	181861	284992	221534

### Pan-core and phylogenetic analysis

**Fig 2:**
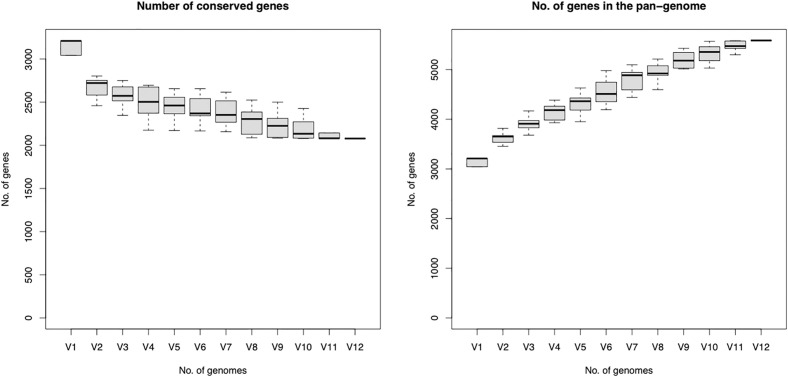
Core and pan genome of *C. kerstersii* strains. Box plots show the change in the number of genes with the sequential addition of genomes from 12 *Comamonas kerstersii* strains. (Left) Number of conserved (core) genes decreases as the additional genomes are incorporated and slowly approaches plateau. (Right) Number of genes in the pan-genome increases as the new genomes add up indicating open pan-genome.

A total of 5586 genes were used from all the strains of *C. kerstersii* (n = 12) for the pan genome analysis, of which 2079 comprised the core genome (99%−100% of strains). Among the accessory genome, 1825 genes were in the shell and 1682 genes were in the cloud. With the introduction of a new genome, the number of core genes declined and hit the plateau (Fig 2). While with each additional genome added, the size of the pan-genome increased steadily suggesting that the *C. kerstersii* has an open pan-genome. The pan-genome indicates that the strains under investigation possess unique genes that are not found in other strains, and that the size of the gene pool will only rise as more genomes are included in the analysis. But coming to such a conclusion should be taken with caution since these kinds of studies should ideally involve more than 100 genome sequences [41].

**Fig 3:**
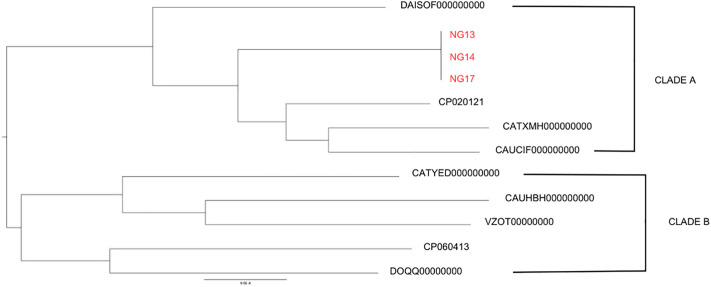
Core-genome phylogenetic tree of *C. kerstersii* strains. Mid-point rooted phylogenetic tree of publicly available C. kerstersii strains and sequenced isolates from Bangladesh shows relationship between the strains based on their core genome alignment. NG13, NG14 and NG17 belonging to CLADE A clustering together indicates their clonal character.

Core genome analysis demonstrated close clustering among the three strains, indicating high genomic similarity and shared evolutionary lineage (Fig 3). The tree shows two distinct clusters – Cluster A and Cluster B. According to the phylogenetic tree, three *C. kerstersii* strains under study - NG13, NG14 and NG17, shared the same ancestor and belonged to Cluster A, with CP020121 being the closest relative. CP020121, also named *C. kerstersii* 8943, was isolated from the effluent of peritoneal dialysis, highlighting the close relationship among human-originated isolates. All these strains shared features like ampicillin resistance and *sul2* and *tetA* genes conferring resistance to sulfonamide and tetracycline, respectively [42].

The CP060413 strain from the other cluster- Cluster B, was isolated from a rectal swab similar to the isolates of our study (Fig 3). Even being from the same clinical source, the CP060413 branched distantly. This strain had no resistance genes other than 2 class A and 1 class C beta-lactamase genes [43].

The *C. kerstersii* strains used in the phylogenetic analysis were isolated from human feces. The presence of various resistance genes across different strains underscores the role of horizontal gene transfer in enabling adaptation to diverse environments.

### Identification of antimicrobial resistance genes (ARG)

Multiple antibiotic resistance genes were identified in all three *C. kerstersii* strains (Table 4). These included genes linked to resistance against β-lactams, aminoglycosides, tetracyclines, and sulfonamides, signifying a varied resistome profile. The *tet(A)* gene, potentially conferred resistance by exporting tetracycline out of the cell in exchange for protons, was identified [44].

**Table 4:** Antimicrobial resistance genes associated with the three strains of *C. kerstersii.*

Strain ID	Resistance Gene	Phenotype	Antibiotic	Coverage (%)	Identity (%)	Alignment Length/Gene Length
NG13, NG14, NG17	aph(6)-Id	Aminoglycoside resistance	streptomycin	100	100	837/837
	aph(3”)-Ib	Aminoglycoside resistance	streptomycin	100	100	804/804
	mph(E)	Macrolide resistance	erythromycin	100	100	885/885
	mph(F)	Macrolide resistance	erythromycin	100	100	900/900
	msr(E)	Macrolide, Lincosamide and Streptogramin B resistance	Erythromycin, azithromycin, Pristinamycin ia, virginiamycin s, quinupristin	100	100	1476/1476
	Sul2	Sulfonamide resistance	sulfamethoxazole	100	100	816/816
	tet(A)	Tetracycline resistance	Doxycycline, tetracycline	100	100	1275/1275

The strains possessed *mph(E), mph(F),* and *msr(E)* genes. The *mph(E)* and *mph(F)* encode macrolide phosphotransferase enzymes that likely inactivate macrolides through phosphorylation [45]. The *msr(E)* gene may confer resistance to macrolides, lincosamides, and streptogramin B [46]. The presence of the *aph(3”)-Ib* and *aph(6)-Id* genes suggests resistance to streptomycin. These genes are often linked and encode enzymes that inactivate streptomycin. The presence of *sul2* gene confer resistance to sulfonamides [47].

We found *macA, macB* and *tolC* encoding for macrolide export protein MacA, macrolide export ATP-binding/permease protein MacB and outer membrane protein TolC, respectively. This MacAB-TolC comprises a tripartite efflux system, contributing to resistance to the macrolide class of antibiotics in *E. coli* and other Gram-negative pathogenic bacteria [48]. Beyond antibiotic resistance, this complex is also involved in the transportation of protoporphyrin [49] lipopolysaccharides [50] and virulence factors, i.e., enterotoxin II [51]. Furthermore, several multidrug resistance protein-encoding genes are also present in the genomes of *C. kerstersii* strains (S1 File).

### Adaptive mechanism and virulence

*C. kerstersii* can adapt to diverse environments, and annotating the genomes revealed some genes that enable them to withstand environmental stressors, including polyphosphate kinase (*ppk*). Polyphosphate (polyP) has previously been linked to osmotic and nutritional stress response in *E. coli* and bacterial growth, survival, and pathogenicity in other bacteria [52,53]. The *C. kerstersii* strains of the study possess *osmY*, a periplasmic protein strongly induced by hyperosmotic stimuli. RAST annotation of the isolates revealed an outer membrane protein A (*ompA*) precursor under the stress response category, also helping to survive a high osmolarity environment [54]. Additionally, a colicin V-producing gene (*cvpA*), crucial for gut colonization, is present in *C. kerstersii* strains [55,56].

Analysis using the Virulence Factor Database (VFDB identified key proteins potentially associated with pathogenicity in all three *C. kerstersii* strains (Table 5). Notable findings include proteins involved with type IV pili (T4P), type II secretion systems (T2SS), type VI secretion system (T6SS), general secretion pathways, and flagella. Both NG13 and NG14 strains encoded proteins involved in T4P biogenesis, including PilB and PilF. Strain NG17 possessed a PilJ protein. T4P plays a crucial role in pilus formation, adhesion, and bacterial motility, all of which contribute to pathogenesis. T4P is important in pilus formation, adhesion, and dynamics [57].

**Table 5:** Virulence profile of *C. kerstersii* strains.

Strain ID	Virulence Factors
NG13, NG14	(pilB, pilF, xcpA/pilD) Type IV pili
NG13	Type II secretion system protein E: General secretory system II, protein E
NG13, NG14	AlgW protein (Alginate regulation)
NG13, NG14, NG17	(tsr) methyl-accepting chemotaxis protein (Flagella)
NG13, NG14	(sugC) ABC transporter, ATP-binding protein SugC
NG13	(tapD/pilD) prepilin peptidase (exe)
NG13, NG14	(tar/cheM) methyl accepting chemotaxis protein II (peritrichous flagella)
NG13, NG14	(xcpR) general secretion pathway protein E (xcp secretion system)
NG13, NG14	(gspE) general secretion pathway protein E (T2SS)
NG13, NG14	(cheD) methyl-accepting chemotaxis protein CheD (peritrichous flagella)
NG14	(sigA/rpoV) RNA polymerase sigma factor
NG14	(tapB) Tap type IV pili
NG17	(clpV1) type VI secretion system AAA+ family ATPase (HSI-I)
NG17	(mgtB) Magnesium transport ATPase, P-type 2 (Mg2 + transport)
NG17	(pilJ) type IV pilus biogenesis protein PilJ (Type IV pili twitching motility related proteins)
NG17	(ECVR50_2114) putative inner membrane ABC-transporter
NG17	(ybtP) ABC transporter ATP-binding/permease rpotein
NG17	(chpA) still frameshift probable component of chemotactic signal transduction system (Type IV pili) (Pseudomonas aeruginosa PAO1)
NG13, NG14, NG17	(CRTR) Cu(I)-responsive transcriptional regulator
	(copZ) Copper chaperone
	(clfA) Multidrug resistance transporter, Bcr/CflA family
	(MO) Multicopper oxidase
	(BCO) Blue copper oxidase CueO precursor
	(CTO) Copper tolerance protein
	(copG) CopG protein
	(CRB) Copper resistance protein B

**Fig 4:**
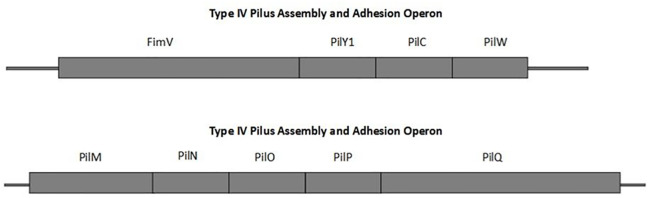
Type IV Pilus Assembly and Adhesion Operons. The schematic diagram illustrates the organization of genes in two Type IV pilus assembly and adhesion operons. The top operon includes genes encoding *FimV*, *PilY1*, *PilC*, and *PilW*, while the bottom operon comprises *PilM*, *PilN*, *PilO*, *PilP*, and *PilQ*. These genes are involved in the assembly and function of the Type IV pilus, a structure critical for bacterial adhesion and motility. Each rectangle represents a gene, with the linear arrangement reflecting their genomic organization.

Genomic analysis of *C. kerstersii* identified two conserved Type IV pilus operons (Fig 4), each encoding essential components for pilus assembly, stability, and function. The first operon (Fig 4), associated with pilus assembly and adhesion, includes genes such as *FimV, PilY1, PilC*, and *PilW*. These proteins are critical for biogenesis, adhesion, and surface colonization [57–59]. The second operon encodes (Fig 4) the core machinery, including PilM, PilN, PilO, PilP, and PilQ, vital for pilus extrusion and stabilization [60]. Interestingly, PilA, the pilin subunit, was absent, indicating possible divergence or alternative pilin expression mechanisms. The presence of these operons underscores their roles in adhesion, motility, horizontal gene transfer, and biofilm formation, highlighting their ecological importance and adaptability in *C. kerstersii* [57–59].

A thorough examination of the *C. kerstersii* genome, revealed several toxin coding sequences (CDS), including zona occludens toxin (zot) protein-encoding sequences that may play virulence-related activities. Other examples are *apxIB, ratA, algC, algE, algE1, clpP and clpP1* (putative Clp proteolytic core), *clpA, clpC*, and *clpX* (Clp-ATPases), and *clpS* (adaptor protein). A cluster of genes involved in copper homeostasis was also found in the annotated genomes of *C. kerstersii* strains (Table 5).

*C. kerstersii* genomes were examined for the protein export and secretion system genes. We found SecA, SecB, and SecYEG encoding genes, although these genes are not present in a cluster. In contrast, TatABC genes encoding the components of the Tat apparatus are present in a cluster (S1 File).

### Plasmids and prophages

In each of the three strains used in this investigation, we located a 157 bp-long fragment identical to plasmids of the IncQ family. 100% identity was found in the region between 5741 and 6536 base pairs (bp) in the plasmid when it was compared using BLAST against the *E. coli* plasmid RSF1010 (Accession no. M28829). The distinct strand-displacement replication mechanism of the IncQ-family of plasmids allows them to work in a broad range of bacterial hosts, and their high mobilization potential enables these plasmids to exhibit great promiscuity [61]. Several studies have mentioned plasmid IncQ to be multidrug-resistant, carrying *tetA, Sul* or carbapenem resistance gene *blaKPC-2* [62,63] although the IncQ1 plasmid of the studied strain does not contain any resistance genes.

**Fig 5:**
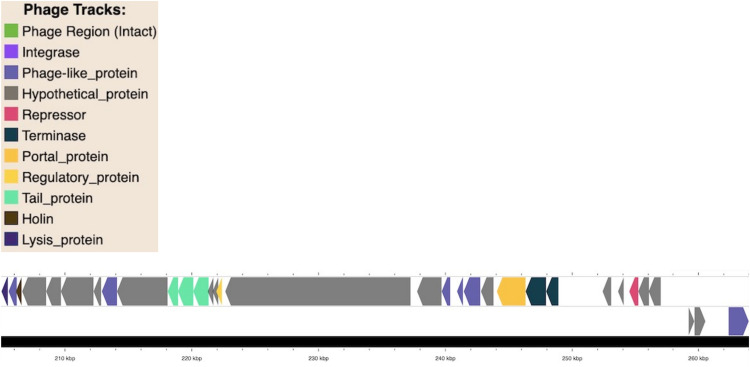
An intact phage region resembling Pseudomonas phage F116. Genomic map of an identified intact prophage region where the arrows indicate the predicted open reading frames (ORFs). Each ORF is color coded according to predicted functional category including holin, phage-like protein, tail protein, lysis proteins. Uncharacterized proteins are depicted in grey.

**Fig 6:**
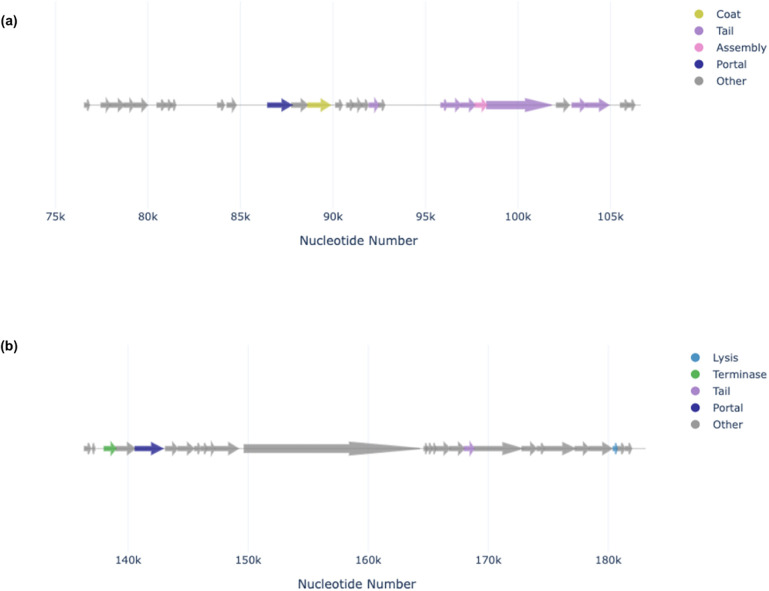
Genomic organization of phage regions belonging to the *Siphoviridae* and *Podoviridae* families. Linear genomic maps of two distinct viral regions with annotated functional genes. **(a)** A phage region belonging to the *Siphoviridae* family with color-coded features highlighting genes encoding coat, tail, assembly, and portal proteins. **(b)** A phage region belonging to the *Podoviridae* family with color-coded features highlighting genes responsible for lysis, terminase, tail, and portal functions. Genes lacking specific functional predictions are classified as “Other” and shown in grey.

Prophage regions were identified in all three *C. kerstersii* strains (Figs 5 and 6). Each prophage region was approximately 63.6 kb in size with a GC content of ~58% and showed similarity to Pseudomonas phage F116 of the Podoviridae family. These regions encoded proteins were involved in phage replication (integrase, repressor, terminases), regulation (regulatory protein), and lysis proteins, tail proteins, holin) and contained a significant number of hypothetical proteins. Interestingly, Phigaro identified an additional prophage region belonging to the *Siphoviridae* family alongside the *Podoviridae* region in each strain (Fig 6a). This region encoded phage assembly proteins (tail, portal, coat proteins). Multiple prophage regions and many hypothetical proteins within them suggest potential roles in *C. kerstersii* fitness. Further investigation of these hypothetical proteins may provide valuable insights into their specific functions.

### *Comamonas kerstersii* host-pathogen PPI network

**Fig 7:**
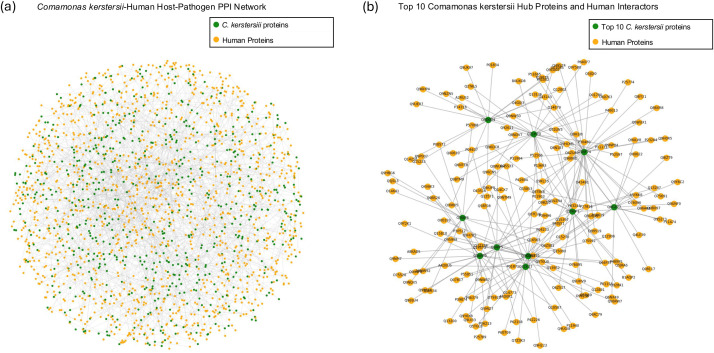
Predicted protein–protein interaction (PPI) network between C. kerstersii and human proteins. (a) Host-pathogen PPI network illustrates predicted interactions where green nodes represent C. kerstersii proteins, while orange nodes represent human proteins. Edges indicate predicted interactions based on sequence similarity to experimentally validated host–pathogen interactions. (b) Subnetwork of the top 10 C. kerstersii hub proteins and their human interaction partners. Green nodes represent the top 10 C. kerstersii proteins with the highest number of interactions. Orange nodes represent human proteins.

Following the BLASTp alignment of clustered *Comamonas* protein sequences with bacterial proteins from the PHISTO database, 1,269 homologous *Comamonas* proteins were identified. These proteins corresponded to known human–bacterial interactions in PHISTO, leading to the prediction of 2,119 protein–protein interactions (PPIs) between *Comamonas* and human proteins (Fig 7a). The PPI network analysis identified the top 10 *Comamonas* hub proteins based on degree centrality. These proteins were Q5NF74, Q7CGS9, Q7CHZ0, Q5NGF1, Q5NFP9, Q8CZZ7, Q8D0D4, Q5NIP6, Q7CKZ7, and Q5NIP5. Together, these hub proteins interacted with 172 unique human proteins. This formed a subnetwork consisting of 184 predicted *Comamonas*–human protein–protein interactions (Fig 7b).

The top 10 *C. kerstersii* hub proteins (Table 6) are involved in a range of functions essential for bacterial survival and interaction with the host. TktA (Q5NF74) and PurC (Q5NGF1) are linked to core metabolic pathways, such as the pentose phosphate pathway and purine biosynthesis, which are important for energy production and nucleotide formation. PgaA (Q8D0D4) is associated with the export of exopolysaccharides, suggesting a role in biofilm formation and surface adhesion. RecN (Q8CZZ7), a DNA repair protein, likely helps the bacteria cope with genomic stress. Two proteins, GatA (Q5NIP6) and GatB (Q5NIP5), function in the transamidation pathway to maintain translation accuracy by correcting mischarged tRNAs—this is particularly important in organisms lacking specific tRNA synthetases. FliN (Q7CHZ0), a component of the flagellar motor switch, supports motility and may aid in navigating the host environment. StbC (Q7CKZ7), which contains a PapC domain, is possibly involved in pilus formation, contributing to host attachment. Another hub, PepP (Q7CGS9), belongs to the peptidase M24B family and may assist in protein degradation or processing. Overall, the identified hub proteins highlight *C. kerstersii'*s reliance on metabolic adaptability, stress response, motility, and host interaction mechanisms—features that may play important roles in its potential pathogenic behavior.

**Table 6:** Functional annotation of top 10 C. kerstersii hub genes.

Serial No.	Uniprot ID	Gene Name	Description
1.	Q5NFP9	typA	GTP-binding protein TypA
2.	Q8D0D4	pgaA	Poly-beta-1,6 N-acetyl-D-glucosamine export porin PgaA
3.	Q7CKZ7	stbC	PapC N-terminal domain
4.	Q8CZZ7	recN	May be involved in recombinational repair of damaged DNA
5.	Q5NGF1	purC	SAICAR synthetase
6.	Q7CHZ0	fliN	Flagellar motor switch protein flin
7.	Q7CGS9	pepP	Belongs to the peptidase M24B family
8.	Q5NF74	tktA	Belongs to the transketolase family
9.	Q5NIP5	gatB	Allows the formation of correctly charged Asn-tRNA(Asn) or Gln-tRNA(Gln) through the transamidation of misacylated Asp- tRNA(Asn) or Glu-tRNA(Gln) in organisms which lack either or both of asparaginyl-tRNA or glutaminyl-tRNA synthetases. The reaction takes place in the presence of glutamine and ATP through an activated phospho-Asp-tRNA(Asn) or phospho-Glu-tRNA(Gln).
10.	Q5NIP6	gatA	Allows the formation of correctly charged Gln-tRNA(Gln) through the transamidation of misacylated Glu-tRNA(Gln) in organisms which lack glutaminyl-tRNA synthetase. The reaction takes place in the presence of glutamine and ATP through an activated gamma-phospho-Glu-tRNA(Gln).

**Fig 8:**
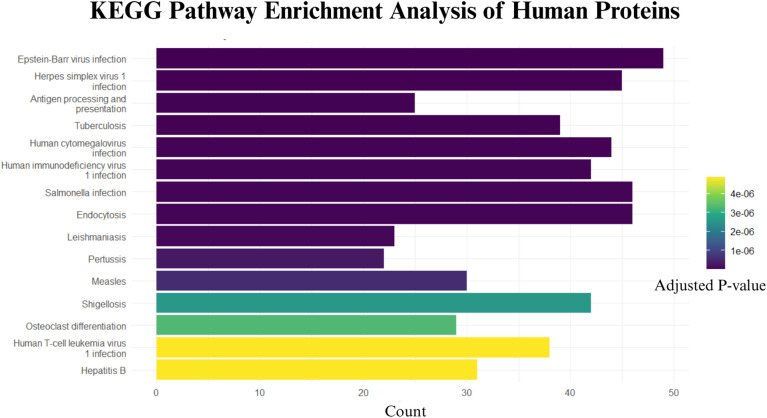
KEGG pathway enrichment analysis of human proteins interacting with *Comamonas kerstersii.* The bar plot shows the top 15 significantly enriched pathways based on adjusted p-values (Benjamini–Hochberg method). The color gradient represents the adjusted p-value, with lighter colors indicating higher significance. Count represents the number of human proteins involved in each pathway.

Enrichment analysis showed significant representation of pathways associated with infectious diseases (Fig 8), including Epstein–Barr virus infection, Herpes simplex virus 1 infection, Human cytomegalovirus infection, and Tuberculosis, suggesting a shared host response mechanism to various pathogens. Notably, Salmonella infection, Shigellosis, and Leishmaniasis were also significantly enriched, highlighting parallels with known bacterial infections. Immune-related pathways such as Antigen processing and presentation, Endocytosis, and Human T-cell leukemia virus 1 infection were prominently represented, indicating a strong involvement of immune signaling in response to the bacterial proteins.

The pathway Hepatitis B showed the strongest statistical significance (lowest adjusted p-value), followed by Human T-cell leukemia virus 1 infection and Osteoclast differentiation, suggesting specific immunological and possibly inflammatory processes may be targeted by the pathogen. The Hepatitis B pathway had the lowest adjusted p-value among the enriched pathways, followed by Human T-cell leukemia virus 1 infection and Osteoclast differentiation. These results indicate statistically supported enrichment of immune-related pathways among the host proteins interacting with *C. kerstersii*. Additionally, the involvement of pathways such as Measles and Pertussis further supports the hypothesis that *C. kerstersii* may engage immune pathways common to viral and bacterial infections.

## Discussion

*C. kerstersii is* becoming a prominent clinical and environmental pathogen due to its broad illness spectrum, ubiquity, adaptability in various environments and resistance capabilities. These characteristics reflect a high degree of genomic and functional diversity, underscoring the need for a deeper understanding of how *C. kerstersii* adapts to diverse ecological niches. This study marks the first identification of *C. kerstersii* in Bangladesh; thus, genomic analysis and understanding the behavior of this bacterium is crucial for mitigating potential public health risks associated with its infections.

In this study, we characterized three *C. kerstersii* isolates (NG13, NG14, and NG17) and compared our results with those from Jiang et al. regarding *C. kerstersii* 8943 [42]. Our isolates show genome sizes of about 3.48 Mb and a GC content of roughly 59.6%, which aligns well with the 3.55 Mb circular genome described by Jiang et al [42]. This similarity indicates that *C. kerstersii* has a relatively compact genome compared to other *Comamonas* species, as previously mentioned [42]. We noticed some significant differences in the antibiotic resistance gene profiles between our isolates and the strain in different studies. While Jiang et al. found genes like *tetA, strB, sul1, blaOXA-1, strA, sul2, catB3,* and *floR*, our isolates carry *aph(6)-Id, aph(3”)-Ib, mph(E), mph(F), msr(E), sul2*, and *tet(A).* The presence *of aph(6)-Id* and *aph(3”)-Ib* suggests a different way of developing streptomycin resistance compared to strA and strB. Other studies back up these findings; one noted resistance to ciprofloxacin [64], and another highlighted resistance to ampicillin [65]. Moreover, a genetic analysis of the *C. kerstersii* strain 3132976 uncovered three β-lactamase genes (CDSs H8N02_05890, H8N02_08740, and H8N02_17110), with H8N02_08740 and H8N02_17110 categorized as class A and C β-lactamases, respectively [66]. Most strikingly, a recent report on strain 121606 indicated resistance to nearly all β-lactam antibiotics, showcasing a broad drug-resistance profile [16]. All in all, these findings underscore the genetic diversity and changing resistance patterns in *C. kerstersii*, highlighting the importance of ongoing monitoring to better inform treatment strategies.

Several studies have mentioned the phylogeny of *C. kerstersii*, for instance, Wu et al., Zhang et al. and Jiang et al. constructed phylogenetic trees [19,42,63], however, the construction of these phylogenies were within the species of the genus *Comamonas* rather than on comparisons among different *C. kerstersii* strains [19,61]. In the present work, we constructed a phylogenetic tree including nine available *C. kerstersii* strains, which revealed clonal relationships alongside evidence of diversity and adaptation to various ecological niches.

One protein of particular interest in conferring niche adaptability is polyphosphate kinase (ppk). The presence of this enzyme explains how *C. kerstersii* is equipped to withstand osmotic stresses in various aquatic environments like blood, urine, or peritoneal fluid. In *V. cholerae*, polyphosphate reserves enhance resistance to environmental stressors, especially under phosphate-limited conditions [67]. The presence of ppk in C. kerstersii suggests a similar mechanism, enabling it to survive in low-phosphate environments (e.g., excrement). PolyP has also been implicated in virulence, including motility and biofilm formation, in a few studies [68–70]. Furthermore, our isolates harbor *osmY,* a periplasmic protein induced by hyperosmotic stimuli, indicating that *C. kerstersii* may tolerate osmotic pressure in environments such as the bladder or colon, as observed in Escherichia coli [71]. The outer membrane protein A (*ompA*) precursor of *C. kerstersii may* enable it to withstand high osmolarity as the protein equips *E. coli* to survive in an acidic environment, high osmolarity, and pooled human serum [54]. OmpA has also been associated with virulence factors like adhesion, invasion, intracellular survival, host defense evasion, and these activities are especially important in the urogenital, respiratory, or nervous system diseases [72]. The inner membrane protein required for strong colonization of the gut is encoded by the *cvpA* gene [55], and the presence of this gene in *C. kerstersii strains* of the study explains its adaptability in the gut of diarrheal patients.

Our three clinical isolates possess additional virulence factors beyond T4P and T6SS. Genes involved in flagellar morphogenesis, assembly, and chemotaxis (S1 File) emphasizes the role of mobility in bacterial pathogenic strategies. To get nutrition and adjust to changing and favorable environmental conditions, bacteria use motility and chemotaxis. In pathogenic bacteria, chemotaxis contributes to host invasion and infection by allowing the bacteria to sense and move towards host tissues and nutrients [73]. Several toxins (S1 File) were also identified in our analysis, including apxIB, which codes for the Toxin RTX-I translocation ATP-binding protein. RTX toxins are known for their rich content of glycine and aspartate, and they are secreted by the type I secretion system found in Gram-negative bacteria [74]. A well-known RTX protein, hemolysin, is produced by the *tlyA* gene in our isolates and has the potential to disrupt the intestinal barrier, leading to symptoms of diarrhea [75]. Additionally, we identified *ratA* gene, which encodes Ribosome association toxin RatA, a component of type II toxin-antitoxin (TA) system. In *E. coli*, the association of RatA protein with the 50S subunit of ribosome prevents the binding of small subunit 30S, thereby preventing the formation of 70S ribosomes, leading to a decrease in protein translation [76].

The *C. kerstersii* strains possess genes that encode proteins involved in the alginate biosynthesis pathway, Phosphomannomutase/phosphoglucomutase, Poly (beta-D-mannuronate) C5 epimerase 1, Poly (beta-D-mannuronate) C5 epimerase 5 encoded by *algC, algE, algE1*, respectively. A negative regulator of alginate synthesis called alginate biosynthesis sensor protein KinB, encoding gene *kinB* is also present in the genome. AlgC protein plays a vital role in producing alginate, LPS and rhamnolipid. Three important *P. aeruginosa* virulence-associated factors Alginate-producing *P. aeruginosa* strains have a mucoid phenotype and are protected from antibiotics and other host defense components [77]. The holin protein present in the phage region can also induce pathogenicity. The enhanced release of extracellular toxins in *E. coli*, including *SheA* and *Stx1*, has been linked to phage-encoded Holins [78]. According to several investigations, holins most likely also have a significant function in the production of biofilms [79]. For instance, it is known that the *Staphylococcus aureus cid* and *lrg* operons regulate cell lysis and the release of genomic DNA, which eventually becomes a structural element of the biofilm matrix and is thus implicated in the production of biofilms [79]. It is also plausible that these strains have acquired virulent characteristics from the other species that may have coexisted in the infection milieu.

Clp protease (caseinolytic protease; ClpP) is a highly conserved serine protease found in many bacteria. This ATP-dependent protease functions as a two-component complex consisting of a regulatory ATPase subunit and a proteolytic subunit. While the proteolytic subunit possesses some intrinsic catalytic activity, both components are essential for optimal enzymatic function in the presence of ATP [80,81]. We identified genes encoding various components of the Clp protease complex, including *clpP* and *clpP1* (putative Clp proteolytic core), *clpA, clpC*, and *clpX* (Clp-ATPases), and *clpS* (adaptor protein). proteases play crucial roles in various cellular processes, including the degradation of misfolded proteins, regulation of short-lived proteins, and housekeeping removal of dysfunctional proteins. They also control cell growth and target DNA-binding proteins from starved cells. Clp*P* has also been linked to the tight regulation of virulence genes in Gram-positive and Gram-negative pathogens such *as Staphylococcus aureus, Listeria monocytogenes* and *Salmonella typhimurium* [80,81]. Therefore, the presence of Clp protease complex encoding genes indicates the contribution of this system to the pathogenic lifestyle of the *C. kerstersii* strains.

We examined the *C. kerstersii* genomes and found that several genes are present to encode enzymes and proteins involved in iron uptake mechanisms. Iron is often limited in host environments as part of the innate immune response, so bacteria must actively scavenge iron from sources like haemoglobin. This iron homeostasis is closely linked to bacterial virulence, stress resistance, and overall fitness. Enterobactin is a siderophore that can extract iron from host iron-binding proteins like transferrin. Haemoglobin-haptoglobin binding proteins allow bacteria to retrieve heme iron from haemoglobin, a major iron source in the host [82]. Iron acquisition regulators like Fur control the expression of iron uptake systems in response to iron availability, allowing bacteria to tightly regulate iron uptake to avoid toxicity while ensuring sufficient iron for growth [82]. All these proteins are key adaptations that enable *C. kerstersii* to efficiently scavenge iron from the host, a critical nutrient for growth and virulence. In addition, several genes encode key enzymes in the heme biosynthesis pathway that is essential for bacterial growth, metabolism, and virulence. Targeting these enzymes could be a potential antimicrobial strategy.

Alternatively, *C. kerstersii* may aid in the infection process through systems not generally linked to virulence but through resistance mechanisms like regulation of copper homeostasis. A cluster of genes involved in copper homeostasis was found in the annotated genomes of the strains (Table 5). Li et al. mentioned that a crucial factor in the pathophysiology of bacteria is copper homeostasis, which involves the complex interaction of bacterial survival strategies and hosts defenses [83]. Host macrophages deliberately create a copper overdose environment to fight invasive pathogens, exposing a host-driven defense mechanism [32]. Copper homeostasis-related genes, such as *copA* (efflux system), *copZ* (copper chaperone), *cueO* (copper oxidase), *copS* (sensor protein), and *copR* (transcriptional activator), were present in all three of the *C. kerstersii* strains included in this study. Notably, studies have shown that loss of the copper efflux pathway (CopA) in certain bacteria, such as *S. pneumoniae*, reduces virulence during infection [84]. This discovery implies that the pathogenic potential of *C. kerstersii* may depend on its capacity to maintain copper homeostasis.

Protein export systems regulate the secretion of different types of proteins across the cell envelope and thus have an important role in colonizing bacteria living in various niches. In *Comamonas* species, the Sec (secretion) and Tat (twin-arginine translocation) pathways are essential for protein transport across the cytoplasmic membrane [85]. The Sec pathway typically handles the translocation of unfolded proteins, whereas the Tat pathway is specialized for transporting fully folded proteins. Most proteins transported by the Sec and Tat pathways remain within the periplasm inner membrane and may be transported in the extracellular environment through type II or type V secretion systems [86]. The genes *epsE, gspF* and out encode components of the type II secretion system and genes to encode the Chaperone-Usher pathway (S1 File). The chaperone–usher (CU) pathway is the most widespread method for assembling adhesive surface pili on the surface of Gram-negative bacteria. For uropathogenic *Escherichia coli* and *Pseudomonas aeruginosa,* these adhesive pili-mediated localization and host colonization and biofilm formation, are considered as important virulence factors [86].

Genomic analysis of *C. kerstersii* has identified two conserved Type IV pilus (T4P) operons crucial to the bacterium's pathogenicity. The first operon is responsible for pilus assembly, adhesion, and surface colonization, and the other operon provides the machinery required for pilus extrusion and stabilization. However, the lack of PilA, the typical pilin subunit, suggests unique evolutionary adaptations. For example, divergence of operon or alternative pilin mechanisms, whereas variability in PilC points to potential strain-specific adaptations. In many Gram-negative pathogens, including *V. cholerae* and *Escherichia coli* type IV pili are widely recognized as critical virulence factors. For *V. cholerae*, the toxin-coregulated pilus (TCP) facilitates adherence to intestinal epithelial cells, which is a vital step in infection and disease development [59]. In *E. coli* O157:H7, hemorrhagic coli pili (HCP) are essential for adherence to host cells, with mutations in the *hcpA* gene, significantly reducing colonization [87]. Identifying these two conserved T4P operons in *C. kerstersii* highlights their pivotal role in adhesion, motility, biofilm formation, and horizontal gene transfer [88], emphasizing their importance in ecological adaptability and pathogenic potential.

The virulence genes, including the toxin proteins zot and RTX, responsible for diarrhea, coupled with the biofilm-producing protein holin, allow *C. kerstersii* to spread infections and fend off host immunological reactions. Although the limited number of samples of *C. kerstersii* is one of the notable limitations of this study and other investigations to fully understand the true nature and genetic diversity of this species. To acquire a comprehensive knowledge of the genetic diversity of this species at the spatial-temporal level and to obtain a more accurate picture of the genetic variety of *C. kerstersii* circulating across the country, it is crucial to increase the number of samples from various sources and places.

The predicted host–pathogen PPI network between *C. kerstersii* and human proteins uncovers mechanisms involved in both host manipulation and bacterial survival. The top 10 hub proteins uncovered metabolic enzymes, DNA repair proteins, motility apparatuses, along with adhesion-related proteins which point toward *C. kerstersii's* reliance on stress tolerance, metabolizing energy, exhibiting motility, as well as interacting with a host, all of which could link to its opportunistic pathogenic capability. Importantly, KEGG pathway enrichment for human interactors had an important overlap with immune-related and infectious disease pathways, which include those implicated in viral infections (e.g., Hepatitis B, EBV, HTLV-1), bacterial infections (e.g., Salmonella, Shigella), and antigen presentation. According to a trend, *C. kerstersii* may exploit host immune responses like other pathogens do. Pathways implicated in inflammatory responses and immune modulation are participating, so *C. kerstersii* may disrupt host defense systems. More research is justified into its role in human disease, especially in hosts with immunocompromised or microbiome disruption.

As a relatively new opportunistic pathogen, *C. kerstersii* has received little attention in scientific research. Only two papers have explored and reported the genomic analysis of this species thus far suggesting that the genomic microbial databases might have incomplete information on *C. kerstersii.* This lack of information may have made it difficult to identify every gene connected to the organism. The presence of many hypothetical proteins has been discovered by the annotated data of the studied strains. These proteins are hypothetical gene products, the roles of which are neither entirely understood nor confirmed by experiments. Even though they are speculative, these potential proteins can provide important information about the genetic composition of *C. kerstersii* and could be useful research targets to clarify their functions in pathogenicity and antibiotic resistance.

## Conclusion

Most researchers focus on well-known pathogens like *V. cholerae* and *E. coli* frequently when isolating microorganisms from diarrheal patients. However, recent advances in high throughput genomics facilities have identified other potential pathogens from diarrheal samples. It is imperative that we adopt state-of-the-art genomics technologies and expand our research focus to consider other possible pathogens like *C. kerstersii.* Significant insights into the role of *C. kerstersii* in diarrheal disorders can be gained from an understanding of its pathophysiology and genetic features. By uncovering previously unknown infections, this widened focus may contribute to developing more thorough diagnostic procedures and focused therapeutic methods. Researchers can improve public health responses and our understanding of diarrheal disorders by investigating the genomes of *C. kerstersii* to identify its virulence factors, resistance mechanisms, and potential epidemiological consequences.

## Supporting information

S1 File Prokka annotated genes of the strains NG13, NG14, NG17. (XLSX)

S2 File Nine publicly available genomes of *C. kerstersii* obtained from (NCBI) used for core-pan genome analysis and phylogenetic tree construction. (XLSX)
